# Diagnosis of chronic obstructive pulmonary disease in lung cancer screening Computed Tomography scans: independent contribution of emphysema, air trapping and bronchial wall thickening

**DOI:** 10.1186/1465-9921-14-59

**Published:** 2013-05-27

**Authors:** Onno M Mets, Michael Schmidt, Constantinus F Buckens, Martijn J Gondrie, Ivana Isgum, Matthijs Oudkerk, Rozemarijn Vliegenthart, Harry J de Koning, Carlijn M van der Aalst, Mathias Prokop, Jan-Willem J Lammers, Pieter Zanen, Firdaus A Mohamed Hoesein, Willem PThM Mali, Bram van Ginneken, Eva M van Rikxoort, Pim A de Jong

**Affiliations:** 1 Radiology, University Medical Center Utrecht, Heidelberglaan 100, 3508 GA Utrecht, The Netherlands; 2 Fraunhofer MEVIS, Universitätsallee 29, 28359 Bremen, Germany; 3 Diagnostic Image Analysis Group, Radiology, Radboud University Nijmegen Medical Centre, Geert Grooteplein 10, 6525 GA, Nijmegen, The Netherlands; 4 Julius Center for Health Sciences and Primary Care, University Medical Center Utrecht, Heidelberglaan 100, 3508 GA, Utrecht, The Netherlands; 5 Image Sciences Institute, University Medical Center Utrecht, Heidelberglaan 100, 3508GA Utrecht, The Netherlands; 6 Radiology, University Medical Center Groningen, Hanzeplein 1, 9700 RB Groningen, The Netherlands; 7 Department of Public Health, Erasmus Medical Center Rotterdam, Dr. Molewaterplein 50, 3015 CE Rotterdam, The Netherlands; 8 Radiology, Radboud University Nijmegen Medical Centre, Geert Grooteplein 10, 6525 GA Nijmegen, The Netherlands; 9 Pulmonology, University Medical Center Utrecht, Heidelberglaan 100, 3508 GA Utrecht, The Netherlands

**Keywords:** Quantitative CT analysis, Computed Tomography, Pulmonary emphysema, Airway remodeling, Lung cancer screening, Chronic obstructive pulmonary disease, Tobacco smoking

## Abstract

**Background:**

Beyond lung cancer, screening CT contains additional information on other smoking related diseases (e.g. chronic obstructive pulmonary disease, COPD). Since pulmonary function testing is not regularly incorporated in lung cancer screening, imaging biomarkers for COPD are likely to provide important surrogate measures for disease evaluation. Therefore, this study aims to determine the independent diagnostic value of CT emphysema, CT air trapping and CT bronchial wall thickness for COPD in low-dose screening CT scans.

**Methods:**

Prebronchodilator spirometry and volumetric inspiratory and expiratory chest CT were obtained on the same day in 1140 male lung cancer screening participants. Emphysema, air trapping and bronchial wall thickness were automatically quantified in the CT scans. Logistic regression analysis was performed to derivate a model to diagnose COPD. The model was internally validated using bootstrapping techniques.

**Results:**

Each of the three CT biomarkers independently contributed diagnostic value for COPD, additional to age, body mass index, smoking history and smoking status. The diagnostic model that included all three CT biomarkers had a sensitivity and specificity of 73.2% and 88.%, respectively. The positive and negative predictive value were 80.2% and 84.2%, respectively. Of all participants, 82.8% was assigned the correct status. The C-statistic was 0.87, and the Net Reclassification Index compared to a model without any CT biomarkers was 44.4%. However, the added value of the expiratory CT data was limited, with an increase in Net Reclassification Index of 4.5% compared to a model with only inspiratory CT data.

**Conclusion:**

Quantitatively assessed CT emphysema, air trapping and bronchial wall thickness each contain independent diagnostic information for COPD, and these imaging biomarkers might prove useful in the absence of lung function testing and may influence lung cancer screening strategy. Inspiratory CT biomarkers alone may be sufficient to identify patients with COPD in lung cancer screening setting.

## Introduction

Computed Tomography (CT)-based lung cancer screening has gained much interest after the National Lung Screening Trial (NLST) reported a 20% lung cancer mortality reduction in the CT arm compared to the chest radiography arm of the screening trial [1]. Although implementation and cost-effectiveness is currently debated, screening has already started after the release of guidelines by two major organizations in the U.S. [2,3]. Lung cancer screening CT scans enable the evaluation of other smoking-induced diseases besides lung cancer, such as chronic obstructive pulmonary disease (COPD), which accounts for significant morbidity and mortality and is also associated with an increased risk for lung cancer. Additional diagnosis of COPD might potentially be useful in the optimization of benefits and cost-effectiveness of CT-based screening in heavy smokers, and may also aid in the identification of the optimal target population for lung cancer screening [4] and personalization of lung cancer screening intervals. However, the opportunity of additional disease evaluation is not yet widely acknowledged [5].

Since lung function testing is not regularly incorporated in lung cancer screening, quantitative imaging biomarkers for COPD are likely to provide important surrogate measurements for disease evaluation. Moreover, computerized quantitative analysis is diagnostically superior to visual scores by human observers [6]. It has recently been shown that automated quantitative analysis of screening CT images may be used to additionally identify COPD among screening participants [7]. In that study, CT emphysema and CT air trapping, together with age, body mass index, smoking status and packyears smoked, provided good accuracy in the identification of COPD. To quantify the extent of air trapping, expiratory CT was added to the lung cancer screening protocol [7].

Software technology now allows for fully automatic analysis of bronchial wall thickness in inspiratory CT [8,9]. Bronchial dimensions of the larger airways on CT have been shown to correlate well with those of the small airways measured by pathology [10], which may suggest that both reflect small airways disease. Another option is that large airway wall thickening is a separate marker of airway disease and visualizes the chronic bronchitis component of COPD [11,12]. Therefore, quantification of bronchial wall thickness may be able to either replace the CT air trapping measurements (and hence the need for additional expiratory imaging), or may add independent diagnostic value for COPD and therewith improve the diagnostic performance of CT. The objective of this study is to determine the independent diagnostic value of CT emphysema, CT air trapping and CT bronchial wall thickness in the identification of COPD in low-dose lung cancer screening CT.

## Materials and methods

This study was performed as a side-study of the Dutch and Belgian lung cancer screening trial (NELSON-trial, ISRCTN 63545820) [13]. The study was approved by both the Dutch Ministry of Health and the local ethical review board of the University Medical Center Utrecht. Each participant provided written informed consent.

### Subjects

The selection of the study population has been described in detail elsewhere [7]. In summary, we included 1140 male subjects with a paired inspiratory and expiratory CT obtained on the same day between July 2007 and September 2008. The included study population was a subsample of the total lung cancer screening population. The subsample did not differ significantly from the total population of subjects screened for lung cancer, as previously reported [7]. Participants included in the lung cancer screening trial were current and former (<10 year) heavy smokers with a smoking history of at least 16.5 packyears, and were at baseline between 50 and 75 years of age. All participants weighted less than 140 kg and were physically fit enough to undergo surgery. Participants provided self-reported presence of respiratory symptoms. Participants were asked to indicate whether cough, sputum production, dyspnea and wheezing were present for at least three months a year. A symptomatic status was assigned when presence of at least 1 of the symptoms was reported. Otherwise, a participant was assigned an asymptomatic status.

### Pulmonary function testing

Prebronchodilator spirometry was performed using ZAN equipment (ZAN Messgeräte GmbH, Germany), according to European Respiratory Society and American Thoracic Society guidelines [14,15]. Forced expiratory volume in one second (FEV_1_) was expressed as percentage predicted. Forced vital capacity was expressed as percentage. We defined COPD as FEV_1_/FVC below 70%, with a classification into mild (FEV_1_ ≥ 80%), moderate (80% > FEV_1_ ≥ 50%) and severe (50% > FEV_1_ ≥ 30%).

### Computed tomography

All subjects received paired inspiratory and expiratory CT, obtained with 16 × 0.75 mm collimation (Brilliance 16P; Philips Medical Systems). Data was acquired according to the low-dose protocol of the lung cancer screening trial; 120 kVp (≤80 kg) or 140 kVp (>80 kg) both at 30 mAs for the inspiratory CT, and 90 kVP (≤80 kg) or 120 kVp (>80 kg) both at 20 mAs for the expiratory CT. The estimated effective dose was at maximum 1.4 millisievert (mSv) for the inspiratory acquisition, and at maximum 0.65 mSv for the expiratory acquisition. CT images were reconstructed from lung bases to apices with slice thickness of 1 mm at 0.7 mm increments, using a smooth reconstruction algorithm (B-filter, Philips).

### Quantitative analysis of emphysema and air trapping

**Figure 1:**
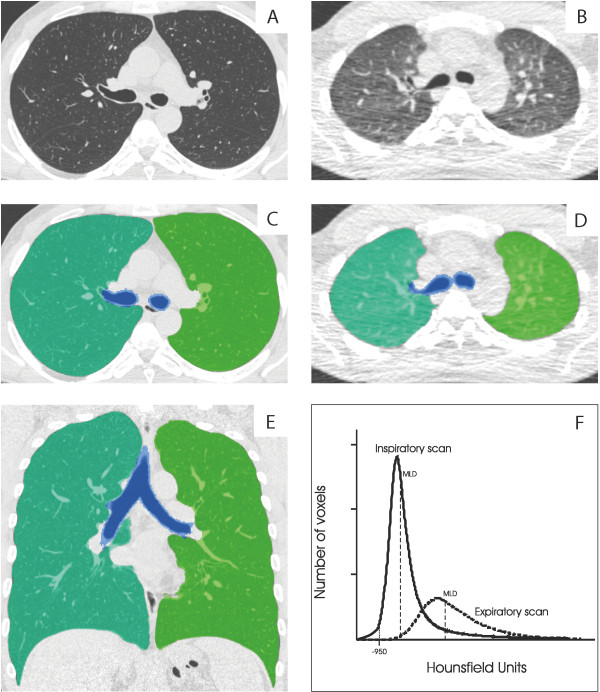
**Illustration of the lung segmentation process and calculation of CT emphysema and CT air trapping. A** = Axial inspiratory CT image; **B** = Axial expiratory CT image; **C** and **D** = Overlay showing the lung segmentation of the right (turquoise) and left (green) lung in an axial slice. The trachea and main bronchi are shown in blue; **E** = Overlay showing the lung segmentation in a coronal inspiratory CT image; **F** = Graph showing the attenuation histograms of both the inspiratory and expiratory CT. CT emphysema is calculated as the percentage of voxels below −950 HU. CT air trapping is calculated as the ratio of the expiratory to inspiratory mean lung density.

The lungs were automatically segmented from the chest wall, mediastinum, airways, vessels and diaphragm using dedicated software [16]. A noise reduction filter was applied to decrease the influence of noise in the low-dose CT images [17]. In both the inspiratory and expiratory images, the attenuation of each voxel in the segmented lung volume was determined and distributed in a density histogram. From these histograms, CT emphysema and CT air trapping were calculated; CT emphysema was defined as the percentage of voxels below −950 Hounsfield Unit (HU) [18]; IN_−950_. CT air trapping was defined as the ratio of expiratory to inspiratory mean lung density [19,20]; E/I-ratio_MLD_. We previously found this to be the most robust measure of air trapping that correlated most closely with lung function [21-23]. Figure 1 illustrates the quantitative assessment of CT emphysema and CT air trapping.

### Quantitative analysis of bronchial dimensions

**Figure 2:**
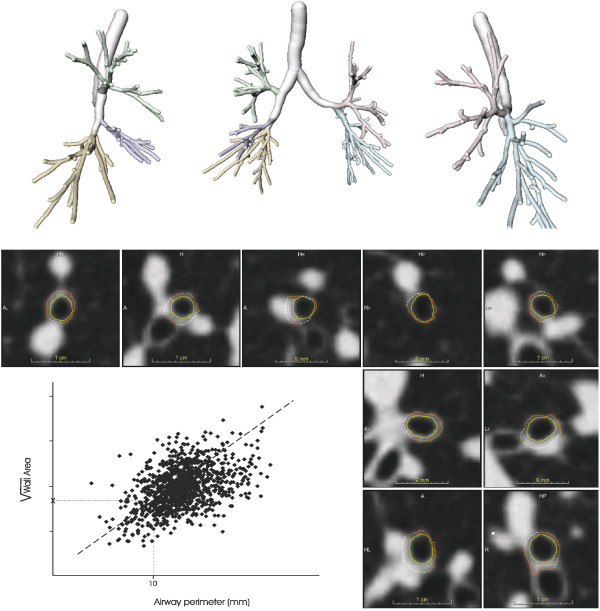
**Illustration of the bronchial segmentation process and the calculation of CT bronchial wall thickness.** The upper part of the figure shows the bronchial tree segmentation of the right and left lung, both separately and combined. The lower part shows a random selection of bronchial cross-sections obtained perpendicular to the bronchial lumen center line. In these bronchial cross-sections, the inner (yellow) and outer (orange) bronchial wall boundaries are shown; solid lines represent observed boundaries whereas dashed lines represent interpolated boundaries. From the observed bronchial wall boundaries the wall area is calculated. The line graph shows a schematic representation of a regression line (dashed line) through the bronchial measurements, from which the square root of wall area for a theoretical bronchial with 10 mm lumen perimeter (i.e. Pi10) was calculated (dotted lines).

The lumen was automatically segmented based on automatic trachea detection and a tree-oriented region growing with multiple optimal thresholds, and converted to a centerline model [8]. Across all centerlines, bronchial cross-sections were defined perpendicular to the local bronchial direction at a 1-mm spacing. Subsequently, the inner and outer bronchial wall boundaries were determined in each of these cross-sections, based on an intensity-integration-based analysis of 72 rays pointing outwards radially from the centerpoint [9]. Cross-sections obtained from the trachea, main bronchi, branching regions as well as cross-sections where the bronchial wall segmentation failed were automatically excluded from further analysis. Using all remaining cross-sections, the regression line of the square root of the wall area versus the lumen perimeter was calculated. The square root of wall area for a theoretical bronchus with 10 mm lumen perimeter (Pi10) was calculated for each subject, which was used as the parameter for bronchial wall thickness [24,25]. Figure 2 illustrates the bronchial segmentation process and the quantitative assessment of CT bronchial wall thickness.

To check the bronchial segmentation process in each case, the 3D bronchial tree segmentation, a random selection of about 90 bronchial cross-sections and the plotted regression line were presented to an independent observer. After carefully reviewing the segmentation results cases with major errors in the bronchial tree segmentation (N = 22) or where segmentation failed (N = 6) were excluded. No manual corrections were performed.

### Statistical analysis

To obtain normal distribution, transformation using the natural logarithm was applied on the CT emphysema measure (logIN_−950_). The other variables did not have to be transformed. Missing values were imputed using multiple imputation technique; quantitative bronchial wall measurements were missing in 28 cases and respiratory symptoms were missing in 55 cases. There were no other variables with missing values.

Various multivariate models were developed using logistic regression analysis with COPD as outcome variable. The modeling started with a baseline model including only demographic variables (i.e. age, body mass index, smoking history and smoking status) as associated variables. Subsequently, quantitative CT emphysema, CT air trapping, and CT bronchial wall thickness were added to the analysis in a stepwise fashion. This resulted in a total of eight different models: 1) demographic variables only; 2) demographic variables + CT emphysema; 3) demographic variables + CT air trapping; 4) demographic variables + CT bronchial wall thickness; 5) demographic variables + CT bronchial wall thickness + CT air trapping; 6) demographic variables + CT emphysema + CT air trapping; 7) demographic variables + CT emphysema + CT bronchial wall thickness; 8) demographic variables + CT emphysema + CT bronchial wall thickness + CT air trapping.

Internal validation was performed for each model by assessing the overoptimism, using bootstrap resampling with 500 iterations [26,27], and subsequent shrinkage of the initial coefficients by the estimated degree of overoptimism [28]. Calibration (i.e. the agreement between the predicted and observed values) of the shrunk models was assessed visually using the calibration plots. Discrimination (i.e. the ability of a model to differentiate between subjects with and without COPD) was assessed by calculation of the area under the receiver operating characteristic (ROC) curve (i.e. C-statistic). The point of optimal accuracy was defined as the highest number of true positive plus true negative cases. For this cut-off value, sensitivity and specificity was calculated for each model. Using the Net Reclassification Index (NRI) [29], all eight models were compared to each other to determine the added value of each quantitative marker. The NRI assesses the added usefulness of a marker by showing the increase in correctly categorized cases (i.e. the shift from false negative to true positive and from false positive to true negative). In addition to the primary analysis in the total study population, we also applied each model separately in the subgroups of symptomatic and asymptomatic subjects.

A p-value lower than 0.05 was considered to represent a statistically significant difference. Analysis were performed using R-statistical program (v.2.13.1) using regression modeling strategies v.3.3-0 and ROCR v.1.0-4 [30]. Values are presented as mean ± SD, unless indicated otherwise.

## Results

Details of the 1140 male lung cancer screening trial participants included in this study are presented in Table 1.

**Table 1:** Study population characteristics

**Characteristic**	**Mean ± SD or Number (%)** **N = 1140**
Age, years	62.5 ± 5.2
Body mass index, kg/m^2^	27.1 ± 3.6
Smoking status	
Current smoker	609 (53.4)
Former smokers	531 (46.6)
Packyears ^*^	38 (28 – 49)
COPD ^†^	437 (38.3)
Mild disease ^‡^	277 (63.4)
Moderate disease ^§^	135 (30.9)
Severe disease ^ll^	25 (5.7)

^*^ Data presented as median (25th – 75th percentile); ^†^ COPD is defined as FEV_1_/FVC < 70%; ^‡^ FEV_1_(% predicted) ≥80%; ^§^ 80% > FEV_1_(% predicted) ≥ 50%; ^ll^ 50% > FEV_1_ (% predicted) ≥ 30%;

FEV_1_ = forced expiratory volume in one second; FEV_1_/FVC = ratio of forced expiratory volume in one second over forced vital capacity.

### Quantitative CT analysis

Segmented lung volumes were 6.79 ± 1.13 liter in inspiratory CT and 3.71 ± 0.90 liter in expiratory CT. The quantified amount of CT emphysema showed a median (P25 – P75) value of 0.75 (0.40 – 1.46)%. For CT air trapping this was 0.84 (0.80 – 0.88). On average 1280 ± 403 (range 260 – 3536) bronchial cross-sections throughout the lung were obtained per subject. The Pi10 for all participants was 2.41 ± 0.51 mm.

### Diagnostic performance of the multivariate models

The baseline model including only demographic variables showed a C-statistic of 0.652. Separately adding the three quantitative CT biomarkers significantly improved the model. The model with CT emphysema showed a C-statistic of 0.784 and an NRI of 22.2%. The model with CT bronchial wall thickness showed a C-statistic of 0.755 and an NRI of 14.7%. The model with CT air trapping showed a C-statistic of 0.773 and an NRI of 24.7%. Details on the reclassification can be evaluated based on the shift in false and true positives and false and true negatives, these data are presented in Table 2.

**Table 2:** Performance measures for the various multivariate models to identify COPD

	**Model**	**TP**	**FN**	**TN**	**FP**	**ACC**	**SENS**	**SPEC**	**PPV**	**NPV**
		**(n)**	**(n)**	**(n)**	**(n)**	**(%)**	**(%)**	**(%)**	**(%)**	**(%)**
1	Baseline model ^*^	125	312	630	73	66.2	28.6	89.6	63.1	66.9
2	+ CT-BWT	185	252	637	66	72.1	42.3	90.6	73.7	71.7
3	+ CT air trapping	317	120	495	208	71.2	72.5	70.4	60.4	80.5
4	+ CT emphysema	229	208	619	84	74.4	52.4	88.1	73.2	74.8
5	+ CT-BWT	237	200	624	79	75.5	54.2	88.8	75.0	75.7
	+ CT air trapping									
6	+ CT emphysema	274	163	618	85	78.2	62.7	87.9	76.3	79.1
	+ CT air trapping									
7	+ CT emphysema	309	128	610	93	80.6	70.7	86.8	76.9	82.7
	+ CT-BWT									
8	+ CT emphysema	320	117	624	79	82.8	73.2	88.8	80.2	84.2
	+ CT-BWT									
	+ CT air trapping									

^*^ Model including age, body mass index, smoking status and packyears of smoking history; TP = true positive; FN = false negative; TN = true negative; FP = false positive; ACC = Accuracy; SENS = Sensitivity; SPEC = Specificity; PPV = Positive predicted value; NPV = Negative predictive value; CT-BWT = Quantitatively assessed bronchial wall thickness.

When a combination of two CT biomarkers was added to the baseline model, diagnostic performance improved even further. Combinations of emphysema and air trapping, emphysema and bronchial wall thickening, or air trapping and bronchial wall thickening showed a C-statistic of 0.834, 0.861 and 0.801, respectively. The NRI compared to the baseline model was 32.4%, 39.9% and 24.8%, respectively.

Ultimately, all three quantitative CT biomarkers were added to the baseline model. In this full model all biomarkers showed independent diagnostic value for COPD. Applying this full model, a further 4.5% of subjects was reclassified into the correct category compared to the model with only inspiratory CT biomarkers. This full model identified 320 of all 437 COPD cases at the cost of 79 false positives, yielding a sensitivity of 73.2% and a specificity of 88.8%. The full model automatically assigned 82.8% of the subjects the correct status. The full model had a C-statistic of 0.873, and compared to the baseline model the full model reclassified 44.4% of the subjects. In the symptomatic subjects the C-statistic of this full model increased to 0.905 [Table 3].

**Table 3:** Discrimination of the various multivariate models in the identification of COPD in subgroups of asymptomatic and symptomatic subjects

	**Model**	**C-index (95% CI)**	
		**Asymptomatics**	**Symptomatics**
1	Baseline model ^*^	0.674 (0.625 -0.722)	0.634 (0.589 - 0.679)
2	+ CT-BWT	0.739 (0.695 - 0.783)	0.764 (0.725 - 0.803)
3	+ CT air trapping	0.737 (0.693 - 0.780)	0.794 (0.759 - 0.829)
4	+ CT emphysema	0.753 (0.707 – 0.800)	0.806 (0.771 - 0.841)
5	+ CT-BWT	0.771 (0.730 – 0.813)	0.821 (0.788 – 0.855)
	+ CT air trapping		
6	+ CT emphysema	0.782 (0.740 - 0.824)	0.872 (0.844 - 0.899)
	+ CT air trapping		
7	+ CT emphysema	0.828 (0.790 – 0.866)	0.886 (0.859 - 0.912)
	+ CT-BWT		
8	+ CT emphysema	0.832 (0.795 – 0.869)	0.905 (0.881 - 0.929)
	+ CT-BWT		
	+ CT air trapping		

^*^ Model including age, body mass index, smoking status and packyears of smoking history; 95% CI = 95^th^ percentile confidence interval; CT-BWT = Quantitatively assessed bronchial wall thickness.

## Discussion

This study shows that CT emphysema, CT air trapping and CT bronchial wall thickness, automatically assessed on low-dose lung cancer screening CT images, all have independent diagnostic value for COPD. The best performance measures were shown by the model that included all three quantitative CT biomarkers. This full model yielded a very good diagnostic performance and assigned the correct status the vast majority of the screening participants. Using the full model almost three-quarter of the COPD subjects in our population were automatically identified, with a low false-positive rate. However, the additional diagnostic value of expiratory CT was limited, and performance measures remained sufficient when expiratory CT was not included.

Our results lead to several considerations. Firstly, although the concept of additional COPD detection has yet received limited attention and early diagnosis of COPD is subject to debate, the concept of identifying COPD as early as possible using screening CT is feasible. The actual extent of benefits from early diagnosis and intensified smoking cessation-strategies is yet unknown, however, it has been shown that early smoking cessation lowers disease progression and decreases COPD-related morbidity and mortality [31,32]. Moreover, in the setting of lung cancer screening there is also a second rationale to diagnose COPD; having COPD or emphysema increases the risk of developing lung cancer and therefore may aid in risk assessment and personalization of the screening strategy [4]. Since lung function testing is generally not included in lung cancer screening, other measurements might be needed to allow for COPD evaluation. Imaging biomarkers as presented in this study may thus prove important surrogate measures in lung cancer screening setting. Notwithstanding, it should be emphasized that the approach of using automated identification of COPD in screening CT cannot yet be widely implemented at this moment as it needs to be externally validated and quantitative CT analyses need further standardization to allow inter-technique and inter-site comparison [33].

Secondly, our results show that inspiratory CT bronchial wall thickness might usefully replace the expiratory CT air trapping measurements. This is specifically interesting given that expiratory acquisitions are generally not performed in other screening trials, and might not be performed when lung cancer screening is actually implemented. The present study shows that the sensitivity and specificity of the diagnostic model remained acceptable when expiratory air trapping was not included into the model, implying that inspiratory CT biomarkers alone are valuable in identifying patients with COPD in early stages in routine lung cancer screening setting. Although addition of the expiratory CT data did reclassify a further 4.5% of subjects, this effect is limited for diagnostic purposes and may not justify obtaining an expiratory CT scan in each screening participant.

Our study has several potential limitations. Firstly, the generalizability of our study may be limited given that our study population comprises only males. Few women are included in the lung cancer screening trial, since there are few women in the Dutch population with accumulated long-term smoking histories [13]. Also, differences in CT protocols (e.g. CT equipment, dose settings, reconstruction algorithm) may influence absolute values obtained from quantitative image analysis. Although we believe it is highly unlikely that such differences could lead to contradicting findings when applied in another study, close attention should always be paid to technical parameters when comparing quantitative results. Secondly, while the official definition of COPD is based on post-bronchodilator spirometry, spirometry in the lung cancer screening trial was obtained without administration of a bronchodilator due to time restrictions. This may lead to some misclassification due to asthma. However, we do not think this is an important confounder given that the prevalence of asthma in a population of heavy smokers is generally very low. In the Netherlands asthma is present in about 2% of all males between 50 and 75 years of age [34].

## Conclusion

In the absence of lung function testing quantitative CT biomarkers provide useful surrogate measures for COPD evaluation, which may in the future influence screening strategy and enhance screening benefits and cost-effectiveness. The present study shows that quantitatively assessed CT emphysema, CT air trapping and CT bronchial wall thickness each contain independent diagnostic information for COPD, and represent independent imaging biomarkers. As the added diagnostic value of expiratory CT air trapping is limited, inspiratory CT biomarkers alone may be sufficient to identify subjects with COPD in lung cancer screening setting.

## Competing interests

Conflicts of interest for the present study: EM van Rikxoort received a grant Netherlands Organisation for Scientific Research (NWO). P Zanen received a grant (EU FP7 grant COPACETIC HEALTH-2007-2.4.5-6).

Conflicts of interest outside the present study: JWJ Lammers received an institutional grant (EU 201379 Copacetic). HJ de Koning received money for Member Advisory Board Roche Diagnostics. M Prokop received institutional grants from Philips and Toshiba Medical Systems. He also received money for lectures or travel expenses from Bracco, CME Science, ESOR, Bayer – Schering, Philips and Toshiba Medical Systems.

## Authors’ contributions

Conception and design of the study was performed by MO RV HJK CA MP JWJL WPThMM BG EMR and PAJ. Data collection was performed by OMM MS II PZ FAAM BG and EMR. Analysis and interpretation of the data was performed by OMM MS CFB MJG EMR and PAJ. The manuscript drafts were written by OMM MS EMR and PAJ. The manuscript was critically revised by CFB MJG II MO RV HJK CA MP JWJL PZ FAAM WPThMM and BG. All authors read and approved the final manuscript.
